# Correlates of Depressive Symptoms among Middle-Aged and Older Homeless Adults Using the 9-Item Patient Health Questionnaire

**DOI:** 10.3390/ijerph17134754

**Published:** 2020-07-02

**Authors:** Lin-Yun Wang, Lan-Ping Lin, Yun-Cheng Chen, Tai-Wen Wang, Jin-Ding Lin

**Affiliations:** 1 Department of Family Studies and Child Development, Shih Chien University, Taipei 104, Taiwan; nose.123@yahoo.com.tw; 2 Department of Senior Citizen Care and Welfare, Ching Kuo Institute of Management and Health, Keelung 203, Taiwan; lanping518@gmail.com; 3 School of Public Health, National Defense Medical Center, Taipei 144, Taiwan; 607810031@mail.ndmctsgh.edu.tw (Y.-C.C.); s8038129@gmail.com (T.-W.W.); 4 Institute of Long-Term Care, Mackay Medical College, New Taipei City 252, Taiwan

**Keywords:** homeless population, depression, mental health, Patient Health Questionnaire-9 (PHQ-9)

## Abstract

This study investigates the screening for depressive symptoms among middle-aged and older homeless adults based on Patient Health Questionnaire-9 (PHQ-9) and examines the possible factors associated with their major depressive symptoms. A cross-sectional survey was employed, and research subjects included 129 homeless people aged 45 years old and over in Taipei Wanhua District and Taipei Main Station. We used a structured questionnaire and face-to-face interview conducted by three social workers to collect data in the analyses. The content of the questionnaire included an informed consent form, demographic characteristics, enabling and need factors of healthcare, and PHQ-9 of homeless people. Results revealed that 15.5% respondents were free of depressive symptoms, 16.3% had mild level (score 5–9), 31.8% had moderate level (score 10–14), 26.4% had moderately severe level (score 15–19), and 10.1% had severe level of depressive symptoms (score 20–27). Adopting a PHQ-9 score 10 as a cut-off point for major depressive symptoms, 68.3% of middle-aged and older homeless adults were the cases needing to be referred to healthcare settings for further recheck in the near future. A multiple regression analysis found gender, age, and usage of psychiatric outpatient care were associated with major depressive symptom occurrence. The female participants were less likely to have major depressive symptoms than the male participants (OR = 0.29, 95% CI = 0.09–0.96). The elderly participants were more likely to have major depressive symptoms than the aged 45–54 years (OR = 5.29, 95% CI = 1.44–19.41). Those participants who have ever used psychiatric outpatient care were significantly more correlated with the occurrence of major depressive symptoms than their counterparts (OR = 3.65, 95% CI = 1.46–9.09). The present study suggests that in the future health policy should eliminate the risk factors of depressive symptoms and improve mental healthcare access, to improve the health and wellbeing of the homeless population.

## 1. Introduction

Homelessness is a serious public health problem driven by social and economic inequality [1], and patients present increasingly complex physical and mental health conditions [2]. Homeless adults were found to have high rates of functional vision diseases, dermatological/hand-foot problems, and tuberculosis prevalence [3]. Hospitalizations among homeless persons are rising [4], and there are significant disparities in in-hospital care and mortality between homeless and non-homeless adults with cardiovascular conditions [5]. The overall healthcare costs for homeless patients were substantial; therefore, it is necessary to treat homelessness as a merge health and social issue to improve the health outcomes of people experiencing homelessness [6].

Individuals who are homeless perceived experiences as negative and unequal in access and use of mainstream primary healthcare services [7]. Low healthcare services utilization and affordability is an issue among the homeless population [8]. Although previous literature provides suggestions on policy and programs to improve the health of homeless individuals, healthcare providers should also need to address influencing factors for homelessness [9]. A South Korean study indicated that service disparities in homeless people exist in terms of the main place to stay, health, and gender and the current homeless support system [10]. In general, homeless adults experienced more unmet healthcare needs than the general population, and policies to eliminate the disparities and barriers of primary care access in these people are needed [11].

The homeless adults tended to have very high burdens of mental health and substance use disorders [12,13]. They were more likely to present, late in their illness and treatment, low utilization of primary care and outpatient services; have high rate of emergency and hospitalization, and poor compliance with medication, and there were less of them in psychiatric services [14]. Those homeless people received a formal diagnosis of mental health conditions, mainly including depression, addiction disorder, anxiety, schizophrenia, and bipolar disorder [15]. According to a previous study, there were 30–50% of homeless people who have a significant mental illness; most prevalent illnesses included functional psychoses, acute distress, and personality dysfunction. Co-morbidity of mental illness and substance abuse occurred in 20%, and they also had a higher rate of physical morbidity than the general population [16]. 

The homeless population is aging, and nearly half of older homeless adults in a population-based cohort became homeless for the first time after age 50 in New York [17,18]. In Ontario, Canada, almost 70 percent of older homeless people reported first becoming homeless between the ages of 41 and 60 [19]. Homeless adults aged 50 and older have unique health problems compared to younger homeless adults [20,21]; the aging trends suggest that chronic conditions will become increasingly prominent for homeless health services [22]. Therefore, there has been a growing focus on the issues of developing strategies to meet the health needs [23] and prevent and end homelessness among older adults [18,24].

There is a strong public stigmatization of homeless people in Taiwan; one previous study indicated that the homeless are not a homogeneous social group but are going through a similar trajectory of street life [25]. The causes of homelessness in Taiwan are somewhat different to those in other developed countries. Typically, a homeless person in Taiwan becomes so through a series of events beyond his control or due to poor decisions that had unforeseen circumstances, such events as unexpected ill-health, loss of his identity card, irreconcilable family conflict, being trapped in some addiction or battling mental challenges, etc. [26]. In Taipei, not everyone had become aware of the homeless issue, due to low housing and basic living coverage and public services, and also there is an inadequate number of social workers responsible for homeless welfare services in the Taipei City Government [27]. To improve homeless services, initiatives such as a universal principle for nationwide homeless delivery systems, employment consultation, and housing compensation, supportive relationships with the mainstream are required [28].

Actions to prevent and respond to homelessness by addressing the mental health problems across the life course are essential [29]. There is a need for public health and social policy efforts to support and care for homeless persons to reduce disparities in healthcare and improve health outcomes for this population and to prevent the elderly people who are at-risk from becoming homeless. Homeless individuals with differing life experiences of different ages may have different needs and strengths and thus need different services and interventions [18]. About one-half of both the chronic and newly homeless aged 50 and older adults have possible or probable depression [19]. As the aging population growing in Taiwan, the middle and older aged people need to pay much attention to the onset of homelessness and the consequent health needs. Especially, there is a significant focus on the mental health of vulnerable populations, but little is known about the profile and correlates of depressive symptoms among homeless persons, particularly in middle-aged and older homeless adults. Therefore, the present study investigates the screening of depressive symptoms based on the Patient Health Questionnaire-9 (PHQ-9) and examines the possible factors associated with their major depressive symptoms.

## 2. Methods

The homeless refers to people who often stay in public places or places where the public can enter, and there were 647 registered homeless people located in Taipei City, Taiwan, according to the official report [30]. In Taipei City, Wanhua District and Taipei Main Station had a higher population of homeless people than other areas. These two areas attract large numbers of homeless people because of the old town history and geographic location in the center of Taipei City. This study employed a cross-sectional survey to recruit homeless people aged 45 years old and over in Taipei Wanhua District and Taipei Main Station, where the study population (N = 292) come from the homeless registries of Wanhua District Welfare Center (*n* = 252) and Homeless Taiwan (*n* = 40). 

Regarding the research ethical considerations, firstly, the Wanhua District Welfare Center and Homeless Taiwan agreed to participate in this study after their official review of the research proposal. It meant the researcher could plan to interview the homeless adults who registered in their service system. Secondly, the researcher introduced the study purposes and right protections to the participant and then they signed informed consent forms. We emphasized that the study was only for academic research and analysis. It was anonymous and the information was strictly confidential, and the questionnaire content or process were not to be disclosed to the public. In the process of filling out the questionnaire, if the participant felt uncomfortable or did not want to answer, they could ask to withdraw freely from this study at any time. Finally, those people who completed the answers to the entire questionnaire received a NT$100 cash coupon for the convenience store in appreciation of their support. 

A structured questionnaire was used and face to face interview conducted by three social workers to collect the data in these two areas—offices of Wanhua District Welfare Center and Homeless Taiwan. Those homeless adults who are literate, communicated in Mandarin or Taiwanese language and could express their needs clearly were included in this study. The interview time was 30 min to 1 h. The content of the Chinese questionnaire included demographic characteristics, enabling (social economic status, living location, medical care setting) and need (health behaviors and health status) factors of healthcare, and depressive symptoms (PHQ-9) of homeless people. The expert surface validity was conducted to improve the readability, consistency, and formatting of the questionnaire. The statistical reliability of the PHQ-9 instrument was assessed using Cronbach alpha with the data collected in this study, and the value was 0.865. 

The PHQ-9 is a nine-question instrument originally given to patients in a primary care setting to screen for the presence and severity of depression; it is widely adopted to test mental health wellbeing [31,32,33] and is suitable for use for patients of various cultural backgrounds [34,35,36,37,38,39,40,41,42]. In the general Chinese population, the Chinese version of the PHQ-9 is a valid tool with good reliability for screening depression [43,44,45], and it is widely used in the populations of China, Hong Kong, and Taiwan [46,47,48]. The items of PHQ-9 are the following: Q1: Little interest or pleasure in doing things? Q2: Feeling down, depressed, or hopeless? Q3: Trouble falling or staying asleep or sleeping too much? Q4: Feeling tired or having little energy? Q5: Poor appetite or overeating? Q6: Feeling bad about yourself—or that you are a failure or have let yourself or your family down? Q7: Trouble concentrating on things, such as reading the newspaper or watching television? Q8: Moving or speaking so slowly that other people could have noticed? Or so fidgety or restless that you have been moving a lot more than usual? Q9: Thoughts that you would be better off dead or thoughts of hurting yourself in some way? As a screening tool for depressive symptoms, the PHQ-9 scores each of the criteria on a scale from ‘‘0’’ (not at all) to ‘‘3’’ (nearly every day), with summed scores ranging from 0 (free of depressive symptoms) to 27 (all symptoms occurring daily). The cut-off points of 5, 10, 15, and 20 represent mild, moderate, moderately severe, and severe depression, respectively [49].

A convenience sampling that recruited data from 129 valid respondents was analyzed by SPSS version 18.0 in this study. Firstly, descriptive analyses were conducted to present data of participant’s characteristics in demographic, enabling and need conditions and their PHQ-9 scores. Secondly, univariate chi-square analyses between depressive symptom scores and the participant’s characteristics were described. Finally, multiple logistic regression models were conducted to identify the associated factors of major depressive symptoms of the participants.

## 3. Results

### 3.1. Demographic, Enabling, and Need Characteristics of Homeless Adults

Table 1 shows the demographic data of the homeless adults: 52.7% subjects were from Taipei City, 21.8% were from new Taipei City, and 15.4% were from other cities. Most of the subjects were male (88.4%), and their average age was 58.3 years. More than 50% of the participants finished junior high school education or less, 51.2% of the subjects were single and 36.4% were divorced. In terms of their current living locations, 41.1% were often located in public parks, 28.7% were in the homeless shelters, and 14% were in train/bus station surroundings. Subjects who experienced less than one year in a homeless condition made up 27.9% of the study population, 41.1% experienced 1–5 years, 15.5% experienced 5–10 years, and 15.5% experienced more than 10 years. 

With respect to enabling factors of the respondents (Table 2), 41.4% expressed that they were unemployed, 14% had a full-time job, and 44.9% of respondents reported that they had a part-time job. Their income sources included job payment (55.1%), disability allowance (11.6), low-income subsidy (3.1%), and other sources, which included family support, charity, and money loan. Nearly 30% of the respondents reported that they had no income at all, 6% got less than 3000 NT$ monthly, 32.6% got 3001–6000 NT$, and only 13.9% respondents expressed that they got more than ten-thousand NT$ monthly. 

The need factors of the respondents are shown in Table 2. Their negative health behaviors showed that 64.3% were smokers, 20.2% have habits of chewing betel nut, and 21.7% often drink alcohol. The National Health Insurance coverage rate was high (95.3%) in people experiencing homelessness, and 79.1% respondents reported that they have usual healthcare settings, and 54.3% respondents accepted healthcare by usual physicians. Regarding healthcare accessibility, approaching healthcare setting by bus (35.7%), walking (24%), and by mass-rapid transit (MRT) (13.2%) were the most commonly used means among the respondents. Most of the respondents (71.3%) were able to complete their healthcare visits in one hour, and 20.9% finished in two hours time. However, 7.8% of cases reported that they had not used any healthcare service in the last year.

### 3.2. Distribution of Depressive Symptoms (PHQ-9 Scores)

Table 3 presents the depressive conditions among middle-and old-aged homeless people. The results showed that 15.5% respondents were free of depression, 16.3% had mild level (score 5–9), 31.8% had moderate depression (score 10–14), 26.4% had moderately severe depression (score 15–19), and 10.1% had severe depression (score 20–27). Adopting a PHQ-9 score 10 as a cut-off point for major depressive symptoms, 68.3% of middle-aged and older homeless adults were the cases needing to be referred to healthcare settings, for further diagnoses and treatments of their depressive conditions.

### 3.3. Correlates of Depressive Symptoms in Homeless Adults

Table 4 shows presents univariate chi-square analyses of the relation between participant’s characteristics and major depressive symptoms (PHQ-9 cutoff point: 10). The results showed that factors of age (*p* = 0.03), gender (*p* = 0.05), and used psychiatric outpatient care (*p* = 0.01) statistically related to the presence of major depressive symptoms, while the other factors did not indicate significant correlation. Finally, the significant factors in univariate tests were put in multiple logistic regression analyses, to identify the associated factors with the occurrence of major depressive symptoms in the participants (Table 5). The results revealed that gender, age, and usage of psychiatric outpatient care were associated with major depressive symptom occurrence. The female participants were less likely to have major depressive symptoms than the male participants (OR = 0.29, 95% CI = 0.09–0.96). The elderly participants were more likely to have major depressive symptoms than the aged 45–54 years (OR = 5.29, 95% CI = 1.44–19.41). Those participants having ever used psychiatric outpatient care were significantly more correlated with the occurrence of major depressive symptoms than their counterparts (OR = 3.65, 95% CI = 1.46–9.09).

## 4. Discussion 

Homelessness aggravates already poor health, as well as susceptibility to worsening health issues such as pneumonia, depression, dementia, and more. The consequence for older homeless people who live on the streets is that they are much more likely to become victims of violent crime because of their weak health and mobility [50]. Older homeless adults are more at risk of developing chronic and debilitating diseases such as diabetes, heart and related respiratory diseases, and others as a result of premature aging [51]. A survey of Oakland’s older homeless, in California, has shown that a large proportion of the older homeless population first became homeless later in life, and once they became homeless, their health declined precipitously [18]. Therefore, specialized healthcare services for the homeless were developed when it became clear that the mainstream healthcare system could not sufficiently address their health needs [52], particularly in the middle-aged and older homeless population whose prevalent chronic diseases meant they needed to make repeated visits to their healthcare provider and adhere to complicated medication regimens, specific diets, and physical routines [53]. The present study was one of the first studies to screen for depressive symptoms and correlates among middle-aged and older homeless adults based on the PHQ-9 tool, to provide information to further the mental health initiative for this group of people.

The cut-off points of PHQ-9 score of 10 has been found to be good in sensitivity and specificity for a screening of major depressive symptoms in primary care settings [32]. Based on this criterion, our results indicated that 68.3% of homeless adults were screened as potential cases (moderate to severe level of depression) and required further diagnosis to identify and follow up their depressive conditions. Compared to other studies, 80% of homeless people reported that they had mental health problems, with 45% having been diagnosed in England [54]. In the USA, approximately 30% of people who are chronically homeless have mental health conditions, over 60% have experienced lifetime mental health problems [55], and nearly half of homeless women meet major depressive disorder criteria, which is double the prevalence in women in general [56]. In Hong Kong, Yim et al. [57] found that the point prevalence of mental illness was 56% in homeless people, and 71% of cases experienced a lifetime history of mental illness. However, quantifying the problems of mental illness among homeless populations is difficult, and estimates have varied considerably according to different approaches [58]. The present study used PHQ-9 scores ≥10 to estimate major depression prevalence; however, PHQ-9 ≥10 may substantially overestimate depressive condition, and there are too many confounding factors needed to control statistically. It has been suggested that the estimation of depression prevalence should be based on further specialist diagnoses to determine cases correctly [59].

Service providers suggested that homelessness among older adults has notably increased, and more awareness and training is needed for members of the public and service providers on psycho-geriatric and health issues/services [19]. Those middle-aged and older homeless who had been continuously homeless have a higher prevalence of disabling conditions and health needs [20,60]. To prevent the middle-aged and older adults from becoming homeless, we should diminish the risk factors of homelessness and health disparities. Homeless individuals are at high risk of poor health and mental illnesses, previous studies disclosing that they tend to be more psychologically distressed than others [61,62,63,64], particularly in depression, addiction disorder, anxiety, schizophrenia, and bipolar disorder [15]. An Australian study [65] found that 31% of homeless people experienced a mental health problem, and nearly half had a mental health problem prior to becoming homeless. In addition, older homeless adults appeared to follow a different treatment trajectory than younger counterparts, possibly because of lower severity of mental illness at baseline, and may need specific interventions to address their unique pathways to homelessness [66].

The participants’ health behaviors showed that 64.3% were smokers, 20.2% have habits of chewing betel nut, and 21.7% often drink alcohol. This population has many physical and mental health problems, and use of drugs, alcohol, and smoking is common, and co-morbidity is common, with a study reporting they have 5.3 ill health conditions per person [15]. In a profile analysis of the health behaviors of homeless people in South Africa, researchers found that homeless patients reported consuming alcohol (32%), smoking cigarettes (33%), and/or using recreational drugs (7%) [67]. As co-morbidity is common among homeless people, to strengthen accessible and available primary healthcare will be a pre-requisite for improving effective health interventions [68], particularly, to enable homeless people to have access to the range of services that already exist, thereby decreasing their need for specialized services, to protect and improve their health [69].

Regarding healthcare accessibility, homeless people face many service disparities, such as a lack of service co-ordination and health insurance as major barriers in service provision [70]. However, the national health insurance coverage is high, and homeless adults can access healthcare facilities easily in Taiwan, and 79.1% of respondents reported that they have usual healthcare settings, and 54.3% of respondents accepted healthcare by usual physicians. Compared to other studies, 57% of the subjects reported that they had a regular source of care and that the barrier factors included male gender, Hispanic ethnicity, and younger age [71]. Depression severity level was significantly associated with medical utilization in homeless individuals [10]. In addition, homeless people with mental health diagnoses and substance abuse were likely to be higher users of healthcare compared to their counterparts [72]. Another study revealed that those homeless people with chronic mental illness and substance dependence were likely to have a regular source of care [71]. However, initiating coordinated treatment programs for homeless adults with mental illness usually results in better health outcomes than usual care [73]. Therefore, how to construct a suitable healthcare system for the older homeless adults is the further direction of health policy initiatives. 

Management of mental illness and substance misuse problems were a challenge to society [12]. The present study found that 38% of respondents had used psychiatric clinic visits, and 14.7% used inpatient services; therefore, healthcare services for this vulnerable population require much more attention. A previous study found that homeless patients had increased rates of emergency care in alcohol and substance abuse and mental-health-related problems [74]. Koegel et al. [75] revealed that 22% of homeless adults met the criteria for chronic mental illness, and less than one-fifth of these cases reported receiving treatment within the last 2 months. Mental health service utilization was influenced by factors of health needs, such as diagnosis, awareness of a mental ill problem. Providing access to primary care physicians and other services in a community-based shelter program can assist in identification of mental illness and cognitive impairment in elderly homeless adults [76]. To improve psychiatric and homeless services, many crucial needs have to be addressed such as understanding these individuals and their lives; being more proactive in helping them; and reinventing long-term, structured, humane residential and inpatient settings [77]. 

Finally, a significant percentage of PHQ-9 issues appeared in the “nearly every day” category and required more attention among this group of people, such as “trouble sleeping”, “feeling bad about yourself”, “feeling tired or having little energy”, “feeling down, depressed, or hopeless”, “trouble concentrating on things”, and “little interest or pleasure in doing things”. The present study also reveals that older homeless adults are more likely to experience major depressive symptoms. In future health policy initiatives, as O’Brien et al. [78] highlight, adopting patient-centered care and targeted interventions can increase primary care access and improve mental health among homeless people [79]. Furthermore, a multisectoral partnership approach, an integrated person-centered care, and initiating a public health system to monitor serious mental illness and health disparities will be beneficious for the homeless population [80].

The many limitations of this study include the cross-sectional design and self-report data lacking a cause-effect relationship, participant’s privacy affecting health status and health behaviors, the recall bias of healthcare utilization, and the low response rate of the participants. Moreover, the PHQ-9 is a screening scale design, rather than a diagnostic tool for depressive symptoms. Therefore, the actual prevalence of depressive symptoms in homeless population is unclear.

## 5. Conclusions

To protect the health rights of homeless people, programs have been targeted to the homeless in general; specific programs have been targeted to certain subpopulations such as middle-aged and older adults that are represented by the nature of their health problems, demographic characteristics that necessitate specialized approaches [69]. The present study revealed that 68.3% of homeless adults were identified as major depressive symptom cases requiring follow-up, particularly in the older age of this population. We conclude that in the future health policy initiatives need to build patient-centered care and initiate effective interventions to improve care accessibility among the homeless [79]. Furthermore, adopting a multisectoral partnership approach to monitor serious mental illness and health disparities will be beneficious for the homeless population. Finally, the present study highlights that the health policy should eliminate the risk factors of depressive symptoms and improve mental healthcare access, to improve the health and wellbeing of the homeless population.

## Figures and Tables

**Table 1:** Middle- and old-aged homeless people demographic characteristics (*n* = 129).

Variable	*n*	%
Gender		
Males	114	88.4
Females	15	11.6
Age, mean ± SD (range)	58.3 ± 7.5 (45.0–83.2)	
45–54 years	34	26.4
55–64 years	66	51.2
≧65 years	29	22.5
Level of education		
Elementary or less	36	27.9
Junior High School	39	30.2
Senior High School	43	33.3
College and above	11	8.6
Marital Status		
Single	66	51.2
Married	6	4.7
Divorced	47	36.4
Others	10	7.7
Current living location		
Convenience store surrounding	1	0.8
Public Park	53	41.1
Main station surrounding	18	14.0
Homeless shelter	37	28.7
Others	20	15.4
Original household location		
Taipei city	68	52.7
New Taipei city	41	31.8
Other cities	20	15.5
Time of Living in the Street		
≦1 year	36	27.9
1–5 years	53	41.1
5–10 years	20	15.5
≧10 years	20	15.5
Smoking status		
Never	46	35.7
Ever	83	64.3
Drinking status		
Never	101	78.3
Ever	28	21.7
Chewing betel nut status		
Never	103	79.8
Ever	26	20.2

**Table 2:** The distribution of enabling and need conditions in middle- and old-aged homeless people (*n* = 129).

Variables	*n* (%)
Employment status	
None	53 (41.1)
Full-time job	18 (14.0)
Part time	58 (44.9)
Economic Source	
Earning income	71 (55.1)
Subsidy for low income	4 (3.1)
Allowance for the disabled	15 (11.6)
Other	39 (30.2)
Current income per month (NT dollars)	
None	38 (29.5)
Less than 3000 dollars	15 (11.6)
3001–6000 dollars	42 (32.6)
6001–9000 dollars	7 (5.4)
9001–10,000 dollars	9 (7.0)
More than 10,000 dollars	18 (13.9)
National health insurance coverage	
No	6 (4.7)
Yes	123 (95.3)
Usual healthcare setting	
No	27 (20.9)
Yes	102 (79.1)
Usual healthcare physician	
No	59 (45.7)
Yes	70 (54.3)
Transportation to clinic visit	
By walking	31 (24.0)
By bus	46 (35.7)
By MRT	17 (13.2)
By motorcycle	8 (6.2)
By bicycle	12 (9.3)
Other	5 (3.9)
Never visit in one year	10 (7.7)
Time spent on clinic visits	
Less than one hour	92 (71.3)
More than one hour	27 (20.9)
Never visit in one year	10 (7.8)
Psychiatric outpatient care	
Never	80 (62%)
Ever	49 (38%)
Psychiatric inpatient care	
Never	110 (85.3%)
Ever	19 (14.7%)

**Table 3:** Depressive symptoms among middle- and old-aged homeless people.

Depressive Conditions	*n* (%)	Mean ± SD
Depression severity ^a^		12.1 ± 6.5
No	20 (15.5)	
Mild	21 (16.3)	
Moderate	41 (31.8)	
Moderately severe	34 (26.4)	
Severe	13 (10.1)	
Major depressive symptom ^b^		
No	41 (31.8)	
Yes	88 (68.2)	

^a^ Depression severity category (PHQ-9 score): no (0–4), mild (5–9), moderate (10–14), moderately severe (15–19), severe (20–27); ^b^ Cut-off point of major depressive symptom: yes (PHQ-9 score ≧ 10), no (PHQ-9 score < 10).

**Table 4:** Relation of homeless people characteristics and major depressive symptoms (chi-square test).

Variables	Major Depressive Symptom ^a^			*p* Value
	Yes; *n* (%)	No; *n* (%)	Total; *n* (%)	
Gender				0.05
Males	81 (71.1)	33 (28.9)	114 (88.4)	
Females	7 (46.7)	8 (53.3)	15 (11.6)	
Age				0.03
45–54 years	19 (55.9)	15 (44.1)	34 (26.3)	
55–64 years	44 (66.7)	22 (33.3)	66 (51.2)	
≧65 years	25 (86.2)	4 (13.8)	29 (22.5)	
Education level				0.42
Elementary or less	28 (77.8)	8 (22.2)	36 (27.9)	
Junior High School	27 (69.2)	12 (30.8)	39 (30.2)	
Senior High School	26 (60.5)	17 (39.5)	43 (33.4)	
College and above	7 (63.6)	4 (36.4)	11 (8.5)	
Marital status				0.29
Single	44 (66.7)	22 (33.3)	66 (51.1)	
Married	2 (33.3)	4 (66.7)	6 (4.7)	
Divorced	35 (74.5)	12 (25.5)	47 (36.4)	
Others	7 (70.0)	3 (30.0)	10 (7.8)	
Current living location				0.64
Convenience store	1 (100)	0	1 (0.8)	
Public park	39 (73.6)	14 (26.4)	53 (41.1)	
Main station	11 (61.1)	7 (38.9)	18(14.0)	
Homeless shelter	23 (62.2)	14 (37.8)	37 (28.6)	
Others	14 (70.0)	6 (30.0)	20 (15.5)	
Original household location				0.92
Taipei city	47 (69.1)	21 (30.9)	68 (52.7)	
New Taipei city	27 (65.9)	14 (34.1)	41 (31.8)	
Other city	14 (70.0)	6 (30.0)	20 (15.5)	
Time of living in the street				0.39
Less than one year	21 (58.3)	15 (41.7)	36 (27.9)	
1–5 year	40 (75.5)	13 (24.5)	53 (41.1)	
5–10 year	13 (65.0)	7 (35.0)	20 (15.5)	
More than ten years	14 (70.0)	6 (30.0)	20 (15.5)	
Smoking status				0.81
Never	32 (69.6)	14 (30.4)	46 (35.7)	
Ever	56 (67.5)	27 (32.5)	83 (64.3)	
Drinking status				0.61
Never	70 (69.3)	31 (30.7)	101 (78.3)	
Ever	18 (64.3)	10 (35.7)	28 (21.7)	
Chewing betel nut status				0.55
Never	69 (67.0)	34 (33.0)	103 (79.8)	
Ever	19 (73.1)	7 (26.9)	26 (20.2)	
Psychiatric outpatient care				0.01
Never	48 (60.0)	32 (40.0)	80 (62.0)	
Ever	40 (81.6)	9 (18.4)	49 (38.0)	
Psychiatric inpatient care				0.11
Never	72 (65.5)	38 (34.5)	110 (85.3)	
Ever	16 (84.2)	3 (15.8)	19 (14.7)	

^a^ Depression symptom cut-off point: yes (≧10), no (<10).

**Table 5:** Logistic regression analysis of major depressive symptom occurrence (*n* = 129).

Variables	OR (95% CI)	*p* Value
Gender		
Males	Reference	
Females	0.29 (0.09–0.96)	0.04
Age		
45–54 years	Reference	
55–64 years	1.40 (0.58–3.43)	0.46
≧65 years	5.29 (1.44–19.41)	0.01
Psychiatric outpatient care		
Never	Reference	
Ever	3.65 (1.46–9.09)	<0.01
